# Bis(l-leucinium) hexa­chlorido­stannate(IV) dihydrate

**DOI:** 10.1107/S2414314625006765

**Published:** 2025-08-15

**Authors:** Rochdi Ghallab, Amina Kemmouche, Mehdi Boutebdja, Stéphane Golhen

**Affiliations:** a Laboratoire de Technologie des Matériaux Avancés Ecole Nationale Polytechnique de Constantine Algeria; b Ecole Nationale Superieure de Biotechnologie de Constantine, Algeria; c Univ Rennes CNRS ISCR (Institut des Sciences Chimiques de Rennes), 35042 Rennes France; University of Aberdeen, United Kingdom

**Keywords:** crystal structure, X-ray diffraction, l-leucine, hexa­chloro­stannate(IV)

## Abstract

The title compound, (C_6_H_14_NO_2_)_2_[SnCl_6_]·2H_2_O, features l-leucinium cations adopting extended conformations, which maximizes the separation between the methyl groups [–CH(CH_3_)_2_] and the polar NH_3_^+^ and COOH moieties. Additionally, an intra­molecular hydrogen bond between the ammonium (NH_3_^+^) group and the carboxyl group induces a slight reduction in the C—C—N bond angles, with an average value of 106.5°, compared to the ideal tetra­hedral angle of 109.5°. The NH_3_^+^ group is nearly coplanar with the C—C—C—C carbon chain in both fragments, whereas the carboxyl (COOH) group and the methyl group at the C5 position deviate significantly from this plane. The octa­hedral complex anion is close to regular. In the crystal, an extensive network of hydrogen bonds links the components into a three-dimensional network.

## Abstract 2

The l-leucinium cations in the title compound adopt extended conformations that maximize the separation between the methyl groups [–CH(CH_3_)_2_] and the polar NH_3_^+^ and COOH groups.

## Graphical abstract

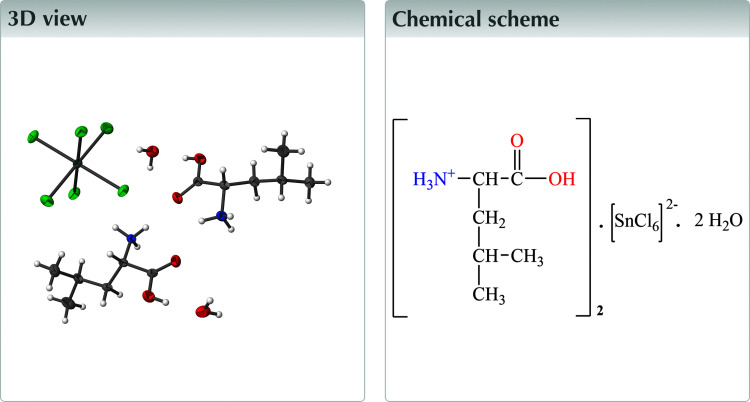

## Structure description

**Figure 1:**
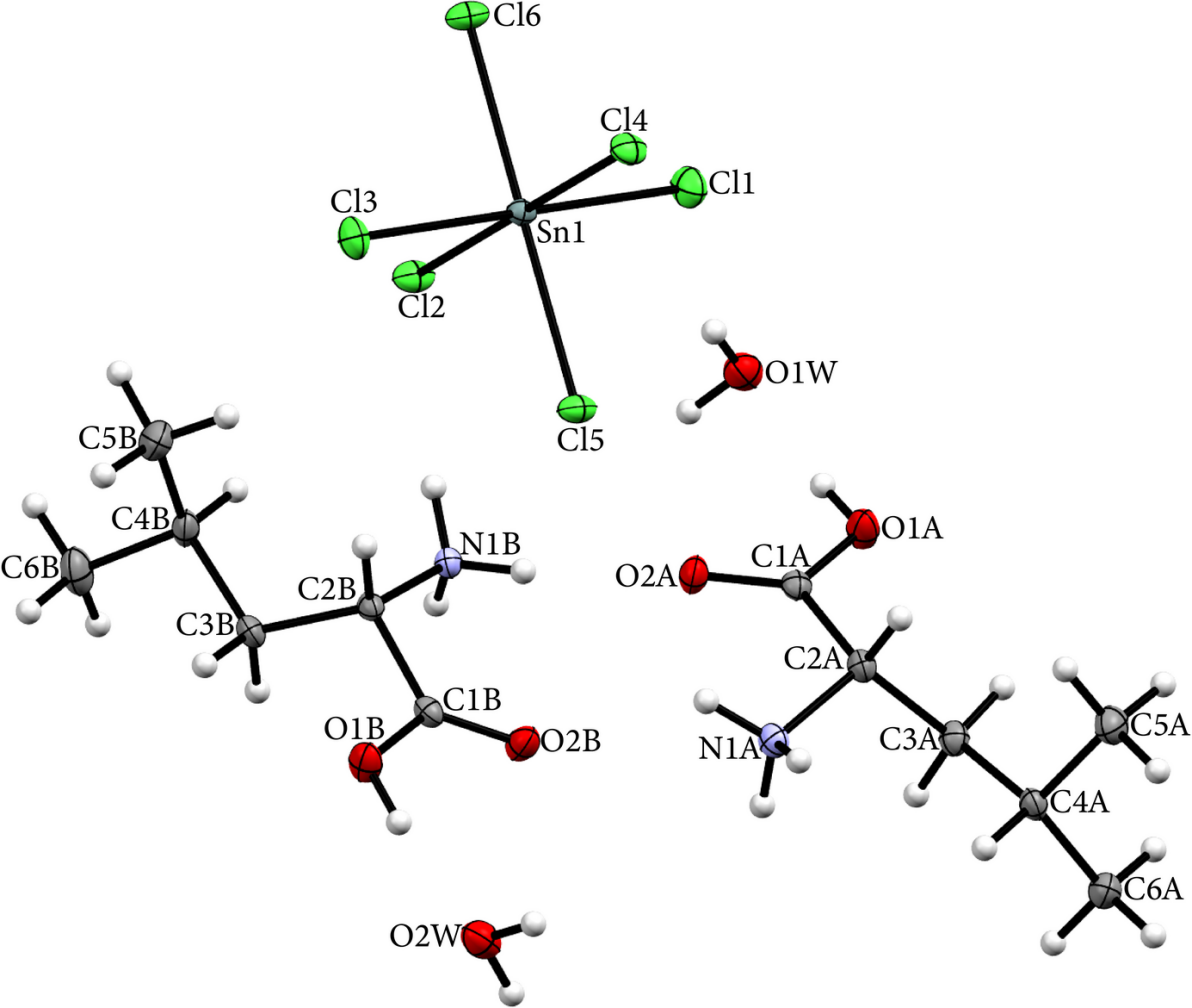
The mol­ecular structure of the title compound with displacement ellipsoids drawn at the 50% probability level.

The title compound, 2(C_6_H_14_NO_2_)^+^·[SnCl_6_]^2–^·2H_2_O, crystallizes in the monoclinic space group *P*2_1_. The asymmetric unit consists of two protonated l-leucinium cations, one hexa­chloro­stannate(IV) anion, and two water mol­ecules of crystallization (Fig. 1 ▸). Equivalent atoms in the cations are labelled C1*A* and C1*B*, *etc*.

During synthesis, the oxidation state of tin atom changed from +II to +IV, resulting in a tin(IV) atom hexa­coordinated by chloride ions and forming a slightly distorted octa­hedral geometry. The Sn—Cl bond lengths range from 2.4045 (11) to 2.4387 (11) Å, while the Cl—Sn—Cl angles deviate by approximately ±1° [88.62 (4)–91.38 (4)°] from the ideal 90° of a regular octa­hedron, indicating only minimal angular distortion. The absence of more significant distortions can likely be attributed to the fact that the hexa­chloro­stannate(IV) anions are discrete; nevertheless, they accept numerous N—H⋯Cl and O—H⋯Cl hydrogen bonds from the organic cations and water mol­ecules, as seen in related structures (Ghallab *et al.*, 2020 ▸; Gheribi *et al.*, 2022 ▸).

The l-leucinium cations in the title compound adopt extended conformations, maximizing the separation between the methyl groups [–CH(CH_3_)_2_] and the polar NH_3_^+^ and COOH groups. This arrangement results in C1—C2 bond lengths that are slightly longer than the median value typically observed for a single C—C bond, with measured values of 1.521 (6) and 1.517 (7) Å for the two cations. The C2—C3—C4 angles, at 115.2 (3) and 115.8 (3)°, are larger than the other C—C—C angles in the carbon backbone (mean: 109.5°), a difference attributed to steric hindrance between the methyl groups and the polar functions. Additionally, an intra­molecular hydrogen bond between the NH_3_^+^ group and the carboxyl group slightly reduce the C1—C2—N1 angles, which average 106.5°, compared to the theoretical tetra­hedral value of 109.5°.

The N atoms of the NH_3_^+^ groups are nearly coplanar with the C2—C3—C4—C6 chains, as indicated by the torsion angles N1—C2—C3—C4 [–68.6 (4) and −62.0 (4)° for the *A* and *B* cations, respectively] and C2—C3—C4—C6 [168.7 (3) and 170.9 (4)°]. In contrast, the COOH group and the methyl group at C5 deviate significantly from this plane, with torsion angles of 170.1 (3) and 178.2 (3)° for C1—C2—C3—C4 and −68.7 (4) and −66.6 (4)° for C5—C4—C3—C2. This extended conformation is consistent with that observed for free l-leucine and its salts with inorganic acids (Zeghouan *et al.*, 2012 ▸; Fleck *et al.*, 2013 ▸; Janczak *et al.*, 2007 ▸), with the notable exception of l-leucinium oxalate (Rajagopal *et al.*, 2003 ▸) and l-leucinium picrate (Anitha *et al.*, 2005 ▸), where the carboxyl group is nearly coplanar with the C2—C3—C4—C6 backbone.

**Figure 2:**
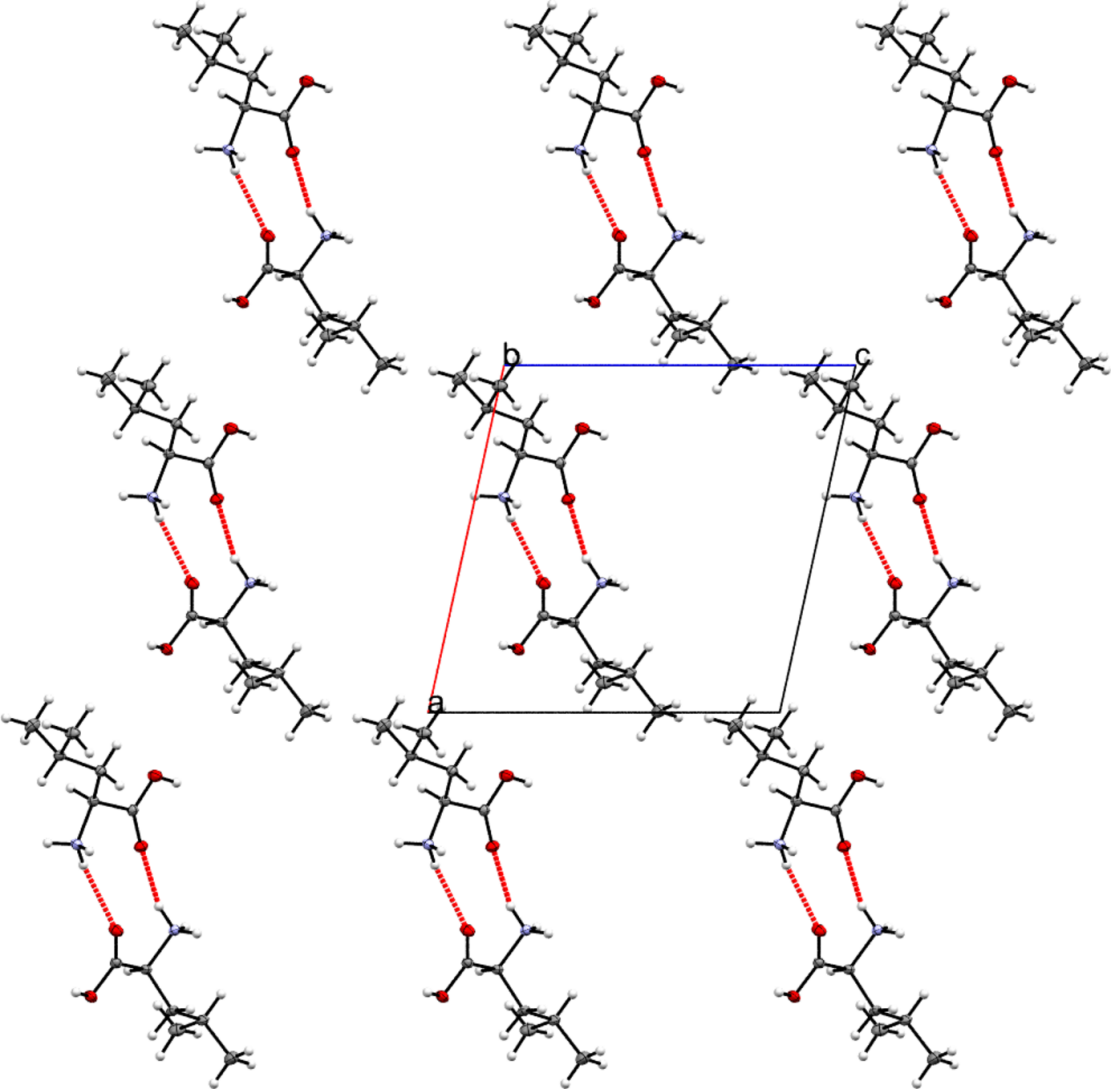
Projection onto the *ac* plane showing 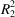
(10) graph-set motifs that organize the mol­ecules into dimers.

The three-dimensional architecture of the extended structure of the title compound is consolidated by an extensive hydrogen-bonding network (Table 1 ▸). A central feature of this network is the 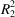
(10) graph-set motif formed by the N1*B*—H1*BA*⋯O2*A* and N1*A*—H1*AC*⋯O2*B* hydrogen bonds (Fig. 2 ▸). This motif organizes the cations into dimers, which propagate along the crystallographic *a*-axis to form hydrogen-bonded layers lying parallel to the *ac* plane.

**Figure 3:**
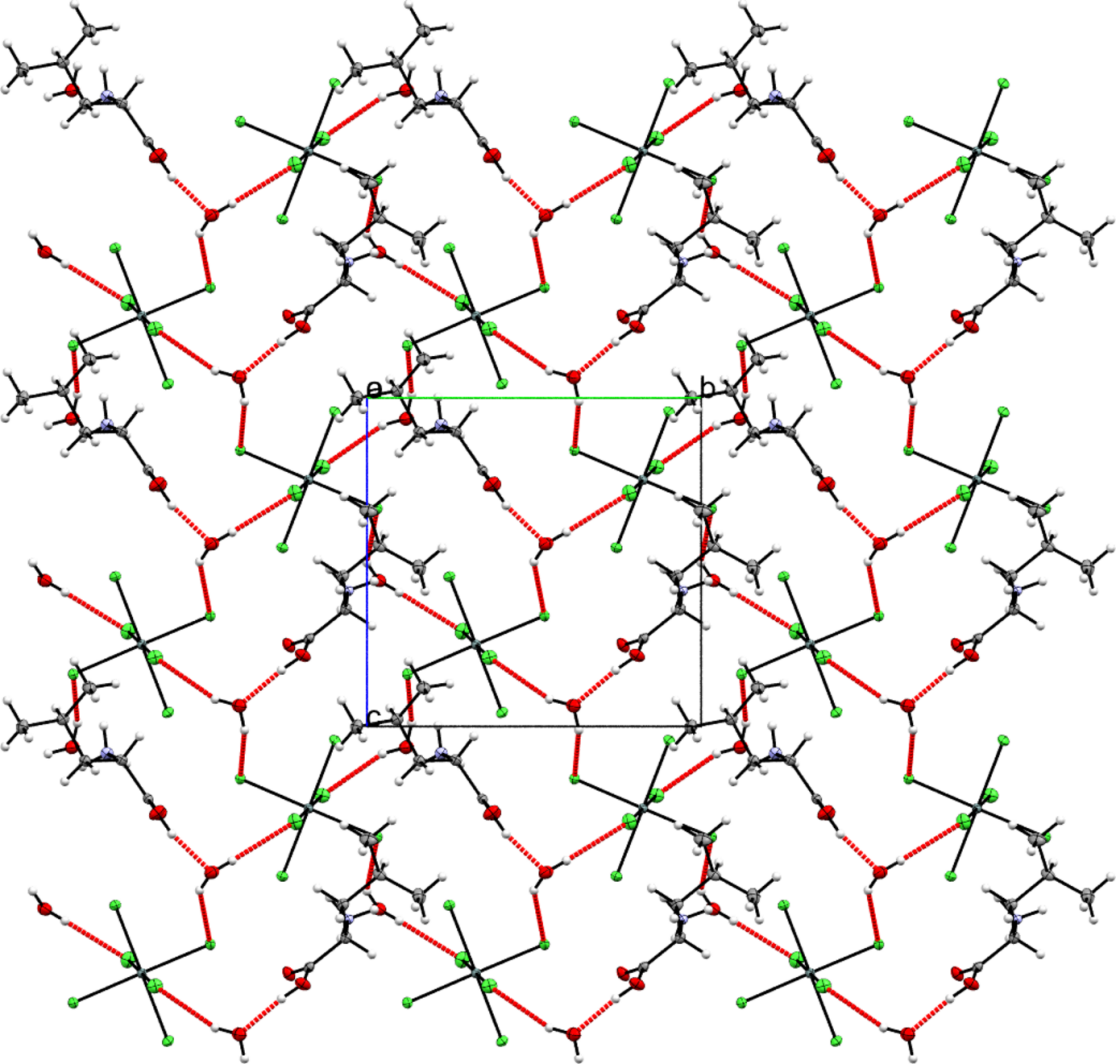
Projection onto the *bc* plane showing the inter­connection of the cationic and anionic sublayers mediated by water mol­ecules.

The water mol­ecules (O1*W*, O2*W*) act as critical structural mediators. Their participation in four key hydrogen bonds, *viz*., O1*W*—H1*WA*⋯Cl6, O1*W*—H1*WB*⋯Cl1, O2*W*—H2*WA*⋯Cl3 and O2*W*—H2*WB*⋯Cl5, anchors the anionic layer. Furthermore, the water mol­ecules bridge the cationic and anionic layers *via* acceptor–donor inter­actions (O1*A*—H1*A*⋯O1*W* and O1*B*—H1*B*⋯O2*W*), effectively inter­connecting the two substructures (Fig. 3 ▸). Additional consolidation arises from N—H⋯Cl hydrogen bonds, which reinforce the cohesion between adjacent layers. These inter­actions, combined with the water-mediated network, create a robust three-dimensional framework.

The synergy between dimer-forming 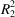
(10) motifs, water-mediated inter­layer connectivity, and N—H⋯Cl inter­actions highlights the hierarchical role of hydrogen bonding in directing the crystal packing. This architecture underscores the importance of solvent mol­ecules in templating anion–cation organization in hybrid inorganic–organic systems.

## Synthesis and crystallization

A mixture of l-leucine (0.262 g) and tin(II) chloride dihydrate (SnCl_2_·2H_2_O, 0.255 g) was dissolved in 20 ml of distilled water acidified with 3 drops of concentrated hydro­chloric acid (HCl, 37%). The solution was stirred and heated at 60°C for 1 h. It was then left to slowly evaporate at room temperature. After 7 days, colourless single crystals suitable for X-ray diffraction analysis were obtained.

## Refinement

Crystal data, data collection and structure refinement details are summarized in Table 2 ▸.

## Supplementary Material

Crystal structure: contains datablock(s) I. DOI: 10.1107/S2414314625006765/hb4529sup1.cif

Structure factors: contains datablock(s) I. DOI: 10.1107/S2414314625006765/hb4529Isup2.hkl

CCDC reference: 2478499

Additional supporting information:  crystallographic information; 3D view; checkCIF report

## Figures and Tables

**Table 1:** Hydrogen-bond geometry (Å, °)

*D*—H⋯*A*	*D*—H	H⋯*A*	*D*⋯*A*	*D*—H⋯*A*
N1*A*—H1*AA*⋯Cl3^i^	0.91	2.78	3.470 (4)	133
N1*A*—H1*AA*⋯Cl5^i^	0.91	2.58	3.342 (4)	142
N1*A*—H1*AB*⋯Cl4^ii^	0.91	2.77	3.471 (4)	134
N1*A*—H1*AB*⋯Cl6^ii^	0.91	2.65	3.452 (4)	148
N1*A*—H1*AC*⋯O2*A*	0.91	2.17	2.620 (5)	110
N1*A*—H1*AC*⋯O2*B*	0.91	2.26	2.959 (5)	133
N1*B*—H1*BA*⋯O2*A*	0.91	1.99	2.873 (5)	164
N1*B*—H1*BA*⋯O2*B*	0.91	2.27	2.626 (5)	103
N1*B*—H1*BB*⋯Cl2	0.91	2.47	3.352 (4)	164
N1*B*—H1*BC*⋯Cl1^iii^	0.91	2.71	3.583 (4)	162
O1*A*—H1*A*⋯O1*W*	0.84	1.79	2.624 (5)	169
O1*B*—H1*B*⋯O2*W*	0.84	1.79	2.627 (6)	173
O1*W*—H1*WA*⋯Cl6^iii^	0.87	2.69	3.440 (3)	145
O1*W*—H1*WB*⋯Cl1	0.87	2.44	3.283 (4)	162
O2*W*—H2*WA*⋯Cl3^ii^	0.87	2.48	3.312 (4)	161
O2*W*—H2*WB*⋯Cl5^i^	0.87	2.63	3.393 (3)	147

Symmetry codes: (i) 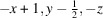; (ii) 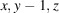; (iii) 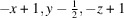.

**Table 2:** Experimental details

Crystal data	
Chemical formula	(C_6_H_14_NO_2_)_2_[SnCl_6_]·2H_2_O
*M*_r_	631.78
Crystal system, space group	Monoclinic, *P*2_1_
Temperature (K)	150
*a*, *b*, *c* (Å)	10.9838 (11), 10.7837 (11), 10.8556 (11)
β (°)	102.316 (4)
*V* (Å^3^)	1256.2 (2)
*Z*	2
Radiation type	Mo *K*α
μ (mm^−1^)	1.68
Crystal size (mm)	0.17 × 0.13 × 0.11

Data collection	
Diffractometer	D8 VENTURE Bruker AXS
Absorption correction	Multi-scan (*SADABS*; Krause *et al.*, 2015 ▸)
*T*_min_, *T*_max_	0.769, 0.831
No. of measured, independent and observed [*I* > 2σ(*I*)] reflections	13707, 5560, 5406
*R*_int_	0.024
(sin θ/λ)_max_ (Å^−1^)	0.649

Refinement	
*R*[*F*^2^ > 2σ(*F*^2^)], *wR*(*F*^2^), *S*	0.021, 0.052, 1.08
No. of reflections	5560
No. of parameters	255
No. of restraints	1
H-atom treatment	H-atom parameters constrained
Δρ_max_, Δρ_min_ (e Å^−3^)	1.14, −0.96
Absolute structure	Flack *x* determined using 2423 quotients [(*I*^+^)−(*I*^−^)]/[(*I*^+^)+(*I*^−^)] (Parsons *et al.*, 2013 ▸)
Absolute structure parameter	−0.004 (10)

Computer programs: *APEX2* and *SAINT* (Bruker, 2014 ▸), *SHELXT2018/2* (Sheldrick, 2015*a* ▸), *SHELXL2019/3* (Sheldrick, 2015*b* ▸) and *OLEX2* (Dolomanov *et al.*, 2009 ▸).

## full crystallographic data

### Bis(L-leucinium) hexachloridostannate(IV) dihydrate Crystal data

**Table:**

(C_6_H_14_NO_2_)_2_[SnCl_6_]·2H_2_O	*F*(000) = 636
*M**_r_* = 631.78	*D*_x_ = 1.670 Mg m^−^^3^
Monoclinic, *P*2_1_	Mo *K*α radiation, λ = 0.71073 Å
Hall symbol: P2yb	Cell parameters from 5406 reflections
*a* = 10.9838 (11) Å	θ = 2.4–27.5°
*b* = 10.7837 (11) Å	µ = 1.68 mm^−^^1^
*c* = 10.8556 (11) Å	*T* = 150 K
β = 102.316 (4)°	Block, white
*V* = 1256.2 (2) Å^3^	0.17 × 0.13 × 0.11 mm
*Z* = 2	

### Bis(L-leucinium) hexachloridostannate(IV) dihydrate Data collection

**Table:**

D8 VENTURE Bruker AXS diffractometer	5406 reflections with *I* > 2σ(*I*)
Radiation source: Enraf Nonius FR590	*R*_int_ = 0.024
CCD rotation images, thick slices scans	θ_max_ = 27.5°, θ_min_ = 1.9°
Absorption correction: multi-scan (SADABS; Krause *et al.*, 2015)	*h* = −14→14
*T*_min_ = 0.769, *T*_max_ = 0.831	*k* = −12→13
13707 measured reflections	*l* = −14→14
5560 independent reflections	

### Bis(L-leucinium) hexachloridostannate(IV) dihydrate Refinement

**Table:**

Refinement on *F*^2^	H-atom parameters constrained
Least-squares matrix: full	*w* = 1/[σ^2^(*F*_o_^2^) + (0.0152*P*)^2^ + 0.9168*P*] where *P* = (*F*_o_^2^ + 2*F*_c_^2^)/3
*R*[*F*^2^ > 2σ(*F*^2^)] = 0.021	(Δ/σ)_max_ = 0.001
*wR*(*F*^2^) = 0.052	Δρ_max_ = 1.14 e Å^−^^3^
*S* = 1.08	Δρ_min_ = −0.96 e Å^−^^3^
5560 reflections	Absolute structure: Flack *x* determined using 2423 quotients [(*I*^+^)-(*I*^-^)]/[(*I*^+^)+(*I*^-^)] (Parsons *et al.*, 2013)
255 parameters	Absolute structure parameter: −0.004 (10)
1 restraint	

### Bis(L-leucinium) hexachloridostannate(IV) dihydrate Special details

**Table:**

Geometry. All esds (except the esd in the dihedral angle between two l.s. planes) are estimated using the full covariance matrix. The cell esds are taken into account individually in the estimation of esds in distances, angles and torsion angles; correlations between esds in cell parameters are only used when they are defined by crystal symmetry. An approximate (isotropic) treatment of cell esds is used for estimating esds involving l.s. planes.
Refinement. Hydrogen atom positions were located in the difference Fourier map, then placed in idealized positions and refined using a riding model, with their displacement parameters set relative to those of their parent atoms. Key experimental parameters and refinement details are provided in the accompanying table.

### Bis(L-leucinium) hexachloridostannate(IV) dihydrate Fractional atomic coordinates and isotropic or equivalent isotropic displacement parameters (Å^2^)

**Table:**

	*x*	*y*	*z*	*U*_iso_*/*U*_eq_	
Sn1	0.49452 (2)	0.82589 (8)	0.24933 (3)	0.01803 (6)	
Cl5	0.50217 (10)	0.62101 (9)	0.15898 (10)	0.0274 (2)	
Cl2	0.58501 (10)	0.74656 (10)	0.45596 (10)	0.0300 (2)	
Cl4	0.40557 (10)	0.90459 (10)	0.04246 (10)	0.0279 (2)	
Cl1	0.28821 (10)	0.78517 (11)	0.28989 (12)	0.0362 (3)	
Cl6	0.48614 (11)	1.03090 (9)	0.33714 (10)	0.0285 (2)	
Cl3	0.70110 (9)	0.86564 (11)	0.21024 (11)	0.0332 (3)	
O2B	0.6272 (3)	0.2663 (3)	0.2471 (3)	0.0309 (7)	
O1B	0.8180 (2)	0.3081 (4)	0.2165 (3)	0.0317 (7)	
H1B	0.805837	0.244024	0.171470	0.048*	
O2W	0.7671 (3)	0.1178 (4)	0.0630 (3)	0.0370 (8)	
H2WA	0.741271	0.045743	0.083037	0.055*	
H2WB	0.721040	0.133722	−0.010916	0.055*	
O1A	0.1824 (3)	0.3717 (3)	0.2622 (3)	0.0356 (9)	
H1A	0.201961	0.416162	0.327006	0.053*	
N1B	0.6272 (3)	0.4429 (4)	0.4137 (3)	0.0211 (7)	
H1BA	0.558359	0.422507	0.354275	0.025*	
H1BB	0.618522	0.521155	0.442085	0.025*	
H1BC	0.636197	0.388650	0.479181	0.025*	
O1W	0.2364 (3)	0.5357 (4)	0.4443 (3)	0.0368 (8)	
H1WA	0.295281	0.499525	0.498906	0.055*	
H1WB	0.267860	0.596358	0.408457	0.055*	
N1A	0.3796 (3)	0.2222 (4)	0.0820 (3)	0.0245 (8)	
H1AA	0.375746	0.212276	−0.001998	0.029*	
H1AB	0.399377	0.148537	0.122200	0.029*	
H1AC	0.438963	0.279448	0.113534	0.029*	
C4A	0.1241 (3)	0.0902 (4)	−0.0217 (4)	0.0235 (9)	
H4A	0.199029	0.069140	−0.056190	0.028*	
O2A	0.3855 (3)	0.3808 (4)	0.2660 (3)	0.0297 (7)	
C1B	0.7214 (3)	0.3273 (7)	0.2674 (3)	0.0214 (7)	
C5B	0.9132 (4)	0.6561 (5)	0.4828 (5)	0.0380 (11)	
H5BA	0.924071	0.725335	0.542663	0.057*	
H5BB	0.843498	0.673945	0.412213	0.057*	
H5BC	0.989605	0.645129	0.451034	0.057*	
C5A	0.0377 (4)	0.1692 (5)	−0.1179 (4)	0.0342 (10)	
H5AA	0.015946	0.124339	−0.198095	0.051*	
H5AB	0.079523	0.247040	−0.130280	0.051*	
H5AC	−0.038261	0.187265	−0.087508	0.051*	
C6B	0.9955 (4)	0.5050 (5)	0.6554 (4)	0.0411 (12)	
H6BA	1.068981	0.487570	0.620531	0.062*	
H6BB	0.974653	0.431433	0.699814	0.062*	
H6BC	1.013350	0.574610	0.714509	0.062*	
C6A	0.0592 (4)	−0.0310 (4)	0.0008 (5)	0.0313 (9)	
H6AA	−0.014605	−0.012152	0.034615	0.047*	
H6AB	0.116771	−0.082361	0.061285	0.047*	
H6AC	0.034027	−0.075865	−0.079050	0.047*	
C3A	0.1681 (3)	0.1569 (4)	0.1045 (3)	0.0240 (7)	
H3AA	0.093960	0.187346	0.133558	0.029*	
H3AB	0.210690	0.095956	0.167339	0.029*	
C4B	0.8860 (4)	0.5380 (5)	0.5489 (4)	0.0274 (9)	
H4B	0.811157	0.552556	0.585628	0.033*	
C2A	0.2556 (3)	0.2656 (4)	0.1022 (3)	0.0219 (7)	
H2A	0.217645	0.319994	0.029534	0.026*	
C3B	0.8591 (3)	0.4281 (4)	0.4578 (3)	0.0235 (7)	
H3BA	0.930140	0.418688	0.415750	0.028*	
H3BB	0.854849	0.351957	0.507577	0.028*	
C2B	0.7402 (3)	0.4372 (3)	0.3568 (3)	0.0198 (7)	
H2B	0.743701	0.514475	0.306735	0.024*	
C1A	0.2819 (3)	0.3453 (5)	0.2205 (4)	0.0206 (10)	

### Bis(L-leucinium) hexachloridostannate(IV) dihydrate Atomic displacement parameters (Å^2^)

**Table:**

	*U*^11^	*U*^22^	*U*^33^	*U*^12^	*U*^13^	*U*^23^
Sn1	0.02014 (10)	0.01690 (10)	0.01714 (9)	0.00183 (9)	0.00416 (6)	0.00103 (6)
Cl5	0.0400 (5)	0.0191 (5)	0.0216 (5)	0.0049 (4)	0.0034 (4)	−0.0014 (4)
Cl2	0.0463 (6)	0.0236 (6)	0.0180 (5)	0.0054 (4)	0.0025 (4)	0.0018 (5)
Cl4	0.0347 (5)	0.0249 (6)	0.0208 (5)	0.0069 (4)	−0.0017 (4)	0.0037 (5)
Cl1	0.0262 (5)	0.0373 (7)	0.0479 (7)	−0.0030 (4)	0.0144 (4)	0.0040 (5)
Cl6	0.0451 (6)	0.0177 (5)	0.0243 (5)	0.0020 (4)	0.0108 (4)	−0.0003 (5)
Cl3	0.0214 (4)	0.0423 (8)	0.0365 (6)	−0.0018 (4)	0.0076 (4)	0.0016 (5)
O2B	0.0273 (14)	0.0302 (17)	0.0378 (17)	−0.0067 (13)	0.0124 (12)	−0.0103 (15)
O1B	0.0242 (12)	0.043 (2)	0.0310 (14)	−0.0022 (13)	0.0118 (10)	−0.0100 (15)
O2W	0.0369 (17)	0.040 (2)	0.0329 (18)	0.0103 (15)	0.0050 (14)	−0.0023 (17)
O1A	0.0240 (13)	0.045 (2)	0.0383 (17)	−0.0010 (12)	0.0077 (12)	−0.0157 (15)
N1B	0.0181 (14)	0.0225 (18)	0.0216 (16)	0.0006 (13)	0.0017 (12)	−0.0006 (15)
O1W	0.0373 (17)	0.040 (2)	0.0320 (18)	0.0051 (15)	0.0046 (14)	−0.0062 (17)
N1A	0.0205 (15)	0.028 (2)	0.0271 (18)	−0.0009 (14)	0.0086 (13)	−0.0004 (16)
C4A	0.0170 (16)	0.028 (2)	0.0248 (19)	0.0019 (14)	0.0029 (14)	−0.0025 (16)
O2A	0.0222 (13)	0.0346 (18)	0.0308 (16)	−0.0059 (12)	0.0022 (12)	−0.0057 (14)
C1B	0.0194 (14)	0.0223 (18)	0.0229 (16)	0.0035 (19)	0.0052 (12)	0.004 (2)
C5B	0.031 (2)	0.033 (3)	0.048 (3)	−0.0072 (18)	0.0033 (19)	−0.004 (2)
C5A	0.032 (2)	0.034 (2)	0.033 (2)	−0.0001 (18)	−0.0029 (17)	0.0020 (19)
C6B	0.028 (2)	0.059 (3)	0.033 (2)	0.001 (2)	−0.0021 (18)	−0.004 (2)
C6A	0.026 (2)	0.029 (2)	0.038 (2)	−0.0026 (17)	0.0049 (17)	−0.0039 (18)
C3A	0.0216 (17)	0.0269 (19)	0.0234 (16)	−0.0024 (14)	0.0042 (13)	0.0026 (15)
C4B	0.0179 (17)	0.035 (2)	0.028 (2)	−0.0027 (16)	0.0015 (14)	−0.0082 (18)
C2A	0.0180 (15)	0.0249 (19)	0.0224 (17)	0.0003 (14)	0.0033 (13)	0.0015 (15)
C3B	0.0169 (15)	0.0259 (19)	0.0264 (17)	0.0040 (13)	0.0016 (14)	0.0011 (14)
C2B	0.0151 (15)	0.0201 (18)	0.0243 (17)	0.0002 (13)	0.0038 (13)	0.0026 (14)
C1A	0.0209 (15)	0.020 (3)	0.0202 (16)	0.0003 (15)	0.0029 (12)	0.0044 (16)

### Bis(L-leucinium) hexachloridostannate(IV) dihydrate Geometric parameters (Å, º)

**Table:**

Sn1—Cl5	2.4259 (13)	O2A—C1A	1.202 (5)
Sn1—Cl2	2.4084 (11)	C1B—C2B	1.517 (7)
Sn1—Cl4	2.4045 (11)	C5B—H5BA	0.9800
Sn1—Cl1	2.4387 (11)	C5B—H5BB	0.9800
Sn1—Cl6	2.4171 (14)	C5B—H5BC	0.9800
Sn1—Cl3	2.4338 (11)	C5B—C4B	1.523 (7)
O2B—C1B	1.206 (6)	C5A—H5AA	0.9800
O1B—H1B	0.8400	C5A—H5AB	0.9800
O1B—C1B	1.313 (4)	C5A—H5AC	0.9800
O2W—H2WA	0.8700	C6B—H6BA	0.9800
O2W—H2WB	0.8700	C6B—H6BB	0.9800
O1A—H1A	0.8400	C6B—H6BC	0.9800
O1A—C1A	1.301 (5)	C6B—C4B	1.522 (6)
N1B—H1BA	0.9100	C6A—H6AA	0.9800
N1B—H1BB	0.9100	C6A—H6AB	0.9800
N1B—H1BC	0.9100	C6A—H6AC	0.9800
N1B—C2B	1.502 (4)	C3A—H3AA	0.9900
O1W—H1WA	0.8704	C3A—H3AB	0.9900
O1W—H1WB	0.8696	C3A—C2A	1.519 (5)
N1A—H1AA	0.9100	C4B—H4B	1.0000
N1A—H1AB	0.9100	C4B—C3B	1.531 (6)
N1A—H1AC	0.9100	C2A—H2A	1.0000
N1A—C2A	1.500 (5)	C2A—C1A	1.521 (6)
C4A—H4A	1.0000	C3B—H3BA	0.9900
C4A—C5A	1.515 (6)	C3B—H3BB	0.9900
C4A—C6A	1.533 (6)	C3B—C2B	1.518 (5)
C4A—C3A	1.532 (5)	C2B—H2B	1.0000
			
Cl5—Sn1—Cl1	91.30 (5)	H5AA—C5A—H5AB	109.5
Cl5—Sn1—Cl3	88.62 (4)	H5AA—C5A—H5AC	109.5
Cl2—Sn1—Cl5	90.58 (5)	H5AB—C5A—H5AC	109.5
Cl2—Sn1—Cl1	89.00 (4)	H6BA—C6B—H6BB	109.5
Cl2—Sn1—Cl6	90.02 (4)	H6BA—C6B—H6BC	109.5
Cl2—Sn1—Cl3	90.58 (4)	H6BB—C6B—H6BC	109.5
Cl4—Sn1—Cl5	89.20 (4)	C4B—C6B—H6BA	109.5
Cl4—Sn1—Cl2	179.56 (5)	C4B—C6B—H6BB	109.5
Cl4—Sn1—Cl1	91.38 (4)	C4B—C6B—H6BC	109.5
Cl4—Sn1—Cl6	90.20 (5)	C4A—C6A—H6AA	109.5
Cl4—Sn1—Cl3	89.04 (4)	C4A—C6A—H6AB	109.5
Cl6—Sn1—Cl5	179.40 (5)	C4A—C6A—H6AC	109.5
Cl6—Sn1—Cl1	88.77 (4)	H6AA—C6A—H6AB	109.5
Cl6—Sn1—Cl3	91.32 (5)	H6AA—C6A—H6AC	109.5
Cl3—Sn1—Cl1	179.57 (6)	H6AB—C6A—H6AC	109.5
C1B—O1B—H1B	109.5	C4A—C3A—H3AA	108.4
H2WA—O2W—H2WB	104.5	C4A—C3A—H3AB	108.4
C1A—O1A—H1A	109.5	H3AA—C3A—H3AB	107.5
H1BA—N1B—H1BB	109.5	C2A—C3A—C4A	115.3 (3)
H1BA—N1B—H1BC	109.5	C2A—C3A—H3AA	108.4
H1BB—N1B—H1BC	109.5	C2A—C3A—H3AB	108.4
C2B—N1B—H1BA	109.5	C5B—C4B—H4B	108.5
C2B—N1B—H1BB	109.5	C5B—C4B—C3B	111.9 (4)
C2B—N1B—H1BC	109.5	C6B—C4B—C5B	110.6 (4)
H1WA—O1W—H1WB	109.5	C6B—C4B—H4B	108.5
H1AA—N1A—H1AB	109.5	C6B—C4B—C3B	108.8 (4)
H1AA—N1A—H1AC	109.5	C3B—C4B—H4B	108.5
H1AB—N1A—H1AC	109.5	N1A—C2A—C3A	111.1 (3)
C2A—N1A—H1AA	109.5	N1A—C2A—H2A	107.9
C2A—N1A—H1AB	109.5	N1A—C2A—C1A	106.5 (3)
C2A—N1A—H1AC	109.5	C3A—C2A—H2A	107.9
C5A—C4A—H4A	108.3	C3A—C2A—C1A	115.4 (3)
C5A—C4A—C6A	110.2 (3)	C1A—C2A—H2A	107.9
C5A—C4A—C3A	112.6 (4)	C4B—C3B—H3BA	108.3
C6A—C4A—H4A	108.3	C4B—C3B—H3BB	108.3
C3A—C4A—H4A	108.3	H3BA—C3B—H3BB	107.4
C3A—C4A—C6A	109.1 (3)	C2B—C3B—C4B	115.8 (3)
O2B—C1B—O1B	125.1 (6)	C2B—C3B—H3BA	108.3
O2B—C1B—C2B	122.5 (3)	C2B—C3B—H3BB	108.3
O1B—C1B—C2B	112.4 (4)	N1B—C2B—C1B	106.5 (3)
H5BA—C5B—H5BB	109.5	N1B—C2B—C3B	111.4 (3)
H5BA—C5B—H5BC	109.5	N1B—C2B—H2B	108.6
H5BB—C5B—H5BC	109.5	C1B—C2B—C3B	113.0 (3)
C4B—C5B—H5BA	109.5	C1B—C2B—H2B	108.6
C4B—C5B—H5BB	109.5	C3B—C2B—H2B	108.6
C4B—C5B—H5BC	109.5	O1A—C1A—C2A	113.3 (3)
C4A—C5A—H5AA	109.5	O2A—C1A—O1A	125.4 (4)
C4A—C5A—H5AB	109.5	O2A—C1A—C2A	121.3 (4)
C4A—C5A—H5AC	109.5		
			
O2B—C1B—C2B—N1B	3.6 (6)	C5B—C4B—C3B—C2B	−66.6 (4)
O2B—C1B—C2B—C3B	126.2 (5)	C5A—C4A—C3A—C2A	−68.7 (4)
O1B—C1B—C2B—N1B	−176.4 (4)	C6B—C4B—C3B—C2B	170.9 (4)
O1B—C1B—C2B—C3B	−53.9 (5)	C6A—C4A—C3A—C2A	168.7 (3)
N1A—C2A—C1A—O1A	−168.7 (4)	C3A—C2A—C1A—O1A	−44.9 (5)
N1A—C2A—C1A—O2A	13.0 (6)	C3A—C2A—C1A—O2A	136.8 (4)
C4A—C3A—C2A—N1A	−68.6 (4)	C4B—C3B—C2B—N1B	−62.0 (4)
C4A—C3A—C2A—C1A	170.1 (3)	C4B—C3B—C2B—C1B	178.2 (3)

### Bis(L-leucinium) hexachloridostannate(IV) dihydrate Hydrogen-bond geometry (Å, º)

**Table:**

*D*—H···*A*	*D*—H	H···*A*	*D*···*A*	*D*—H···*A*
N1*A*—H1*AA*···Cl3^i^	0.91	2.78	3.470 (4)	133
N1*A*—H1*AA*···Cl5^i^	0.91	2.58	3.342 (4)	142
N1*A*—H1*AB*···Cl4^ii^	0.91	2.77	3.471 (4)	134
N1*A*—H1*AB*···Cl6^ii^	0.91	2.65	3.452 (4)	148
N1*A*—H1*AC*···O2*A*	0.91	2.17	2.620 (5)	110
N1*A*—H1*AC*···O2*B*	0.91	2.26	2.959 (5)	133
N1*B*—H1*BA*···O2*A*	0.91	1.99	2.873 (5)	164
N1*B*—H1*BA*···O2*B*	0.91	2.27	2.626 (5)	103
N1*B*—H1*BB*···Cl2	0.91	2.47	3.352 (4)	164
N1*B*—H1*BC*···Cl1^iii^	0.91	2.71	3.583 (4)	162
O1*A*—H1*A*···O1*W*	0.84	1.79	2.624 (5)	169
O1*B*—H1*B*···O2*W*	0.84	1.79	2.627 (6)	173
O1*W*—H1*WA*···Cl6^iii^	0.87	2.69	3.440 (3)	145
O1*W*—H1*WB*···Cl1	0.87	2.44	3.283 (4)	162
O2*W*—H2*WA*···Cl3^ii^	0.87	2.48	3.312 (4)	161
O2*W*—H2*WB*···Cl5^i^	0.87	2.63	3.393 (3)	147

Symmetry codes: (i) −*x*+1, *y*−1/2, −*z*; (ii) *x*, *y*−1, *z*; (iii) −*x*+1, *y*−1/2, −*z*+1.
